# suPAR, a Circulating Kidney Disease Factor

**DOI:** 10.3389/fmed.2021.745838

**Published:** 2021-10-06

**Authors:** Changli Wei, Ryan Spear, Eunsil Hahm, Jochen Reiser

**Affiliations:** Department of Medicine, Rush University Medical Center, Chicago, IL, United States

**Keywords:** suPAR, isoform, proteinuric disease, kidney, uPAR

## Abstract

Urokinase plasminogen activator receptor (uPAR) is a multifaceted, GPI-anchored three-domain protein. Release of the receptor results in variable levels of soluble uPAR (suPAR) in the blood circulation. suPAR levels have been linked to many disease states. In this mini-review, we discuss suPAR as a key circulating molecule mediating kidney disease with a particular focus on differently spliced isoforms.

## The Implication of uPAR in Kidney Disease

The urokinase-type plasminogen activator receptor (uPAR) is a GPI-anchored membrane bound protein involved in many physiological and pathological events. It acts as a receptor for urokinase-type plasminogen activator (uPA), facilitating the generation of activated plasmin, thus playing a role in the directional invasion of migrating cells. It is also implicated in a plethora of cellular responses that include cellular adhesion, differentiation, proliferation and migration in a non-proteolytic fashion as a signaling orchestrator (1, 2). uPAR is a member of the lymphocyte antigen 6 (Ly-6) superfamily proteins, containing three domains, namely DI-DIII, as numbered from the N terminus (3). Protein structure analyses show that uPAR packs into a concave structure with uPA binding to the central cleft, while vitronectin binding to the outside surface (4, 5). This special protein structure makes it possible for uPAR to bind different ligands simultaneously, allowing coordinated regulation of proteolysis, cell adhesion and signaling (6–9). Yet, the structure of the unoccupied human uPAR has not been determined, due to the difficulty in crystalizing the protein (10, 11).

uPAR is expressed on a variety of cells, including monocyte, lymphocyte, endothelial cells (12). The efforts to examine the expression of uPAR in normal kidney and its alterations in kidney disease started in the mid 1990 (13). Almus-Jacobs et al. found the stimulation of murine uPAR gene by endotoxin (14). Xu et al. observed the upregulation of uPAR expression in the glomeruli and in the arterial walls of thrombotic microangiopathy (15). Within the glomeruli of rejected kidney samples, Tang et al. reported positive immunostaining for uPAR in the mesangial cells, but not in the majority of endothelial cells (16). In a rat model of nephrotoxic nephritis, the induction of glomerular uPAR expression was observed as soon as 1 h after nephrotoxic serum injection (17). An unusual implication of uPAR in obstructive nephropathy was reported in unilateral ureteral obstruction (UUO) mouse model, whereby uPAR deficiency accelerated renal fibrosis (18, 19). These findings suggest that renal uPAR may have protective effects in attenuating the fibrogenic response to some renal injury. In the renal biopsy of acute renal allograft rejection, Roelofs et al. found both uPA and uPAR are upregulated (20). Our team identified podocyte uPAR as an important molecule mediating glomerular filtration barrier function in 2007 (21). We found the induction of glomerular uPAR in both human and rodent proteinuric kidney diseases. Gene transfer of uPAR to podocytes but not that to endothelial cells in uPAR deficiency mice induced proteinuria, suggesting the expression of uPAR from podocytes was required for proteinuria development. Mechanistically, uPAR activated αVβ3 integrin in podocytes, promoting cell motility and the activation of small GTPase Rac-1 (21).

## The Implication of suPAR in Proteinuric Kidney Disease

The presence of soluble form of uPAR or generally suPAR was first reported by Ploug et al. (22), when phorbol 12-myristate 13-acetate (PMA)-stimulated U937 cells were treated with bacterial phosphatidylinositol-specific phospholipase-C (PI-PLC). Subsequently, suPAR was detected in many body fluids, such as plasma, serum, urine, saliva, and cerebrospinal fluids. Since then, the elevation of circulating suPAR has been documented in many disease states, reflecting the activation state of the immune system (12).

The initial study of suPAR in proteinuric kidney disease was largely prompted by the concept of a circulating blood factor that causes primary or recurrent focal segmental glomerulosclerosis (FSGS). FSGS refers to a histologic pattern that involves different etiology yet shares a common theme of podocyte injury and/or depletion (23). Generally, FSGS is divided into two categories, primary and secondary. While many studies support the idea that primary FSGS is presumably caused by circulating permeability factor or factors, the identification and characterizing of such factor or factors have been painstakingly challenging. In 2011, our team published the findings that indicate suPAR contributes to primary and recurrent FSGS as a circulating factor (24). suPAR fulfills the criteria of a circulating FSGS factor such as: elevated concentration in patients and the ability to signal to podocytes thereby causing injury and disease. suPAR's injurious activity can be blocked by antibodies against integrin or by lowering suPAR through plasma exchange (25). Some other less characterized candidates for FSGS factor include active proteinases (26), cardiotrophin-like cytokine-1 (27, 28), and protein tyrosine phosphatase receptor O (29).

Using patient samples, we found elevated serum suPAR in two-thirds of primary FSGS, but not in other glomerular disease. In the transplantation subgroup, the mean levels of suPAR, both pre- and post-transplantation, were significantly higher in recurrent FSGS patients than in non-recurrent FSGS patients (24). Like the membrane-bound uPAR, suPAR activated β3 integrin in podocytes. Sustained expression of the secreted form of mouse uPAR induced proteinuria as well as kidney pathological changes in mice (24). In a follow-up study with two independent primary FSGS cohorts, 70 patients from the FSGS clinical trial (CT) and 94 patients from PodoNet, we found the increase of circulating suPAR in 84.3% of CT, 55.3% of PodoNet FSGS patients, compared with 6% of controls (30). Note that CT is a North-America based randomized study that compared the efficacy of cyclosporine A with the combination of mycophenolate mofetil and dexamethasone. Key inclusion criteria are age 2–40 years, eGFR > 40 ml/min per 1.73 m^2^, biopsy-proven FSGS, and resistance to corticosteroid therapy. PodoNet however is a Europe-based consortium for clinical, genetic, and experimental study of SRNS. The inclusion criteria are children (age 0–18 years) with steroid-resistant nephrotic syndrome (SRNS) based on management protocols at the participating medical centers and adults with familial SRNS. The difference in patient population could partially account for the difference in suPAR levels with these two primary FSGS cohorts.

**Figure 1:**
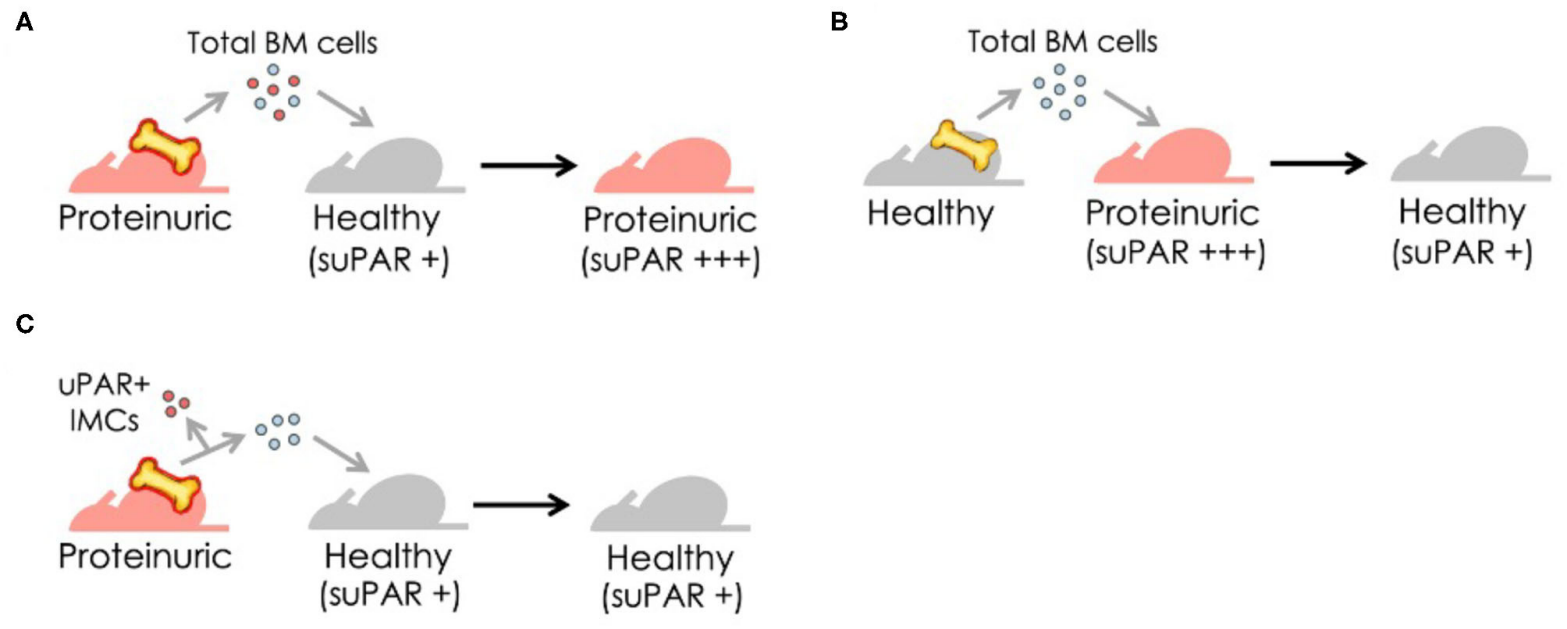
Bone marrow (BM)-derived immature myeloid cells (IMCs), newly identified as a cellular source of suPAR, transfer disease from proteinuric to healthy mice. **(A)** Transfer of BM cells from proteinuric to healthy mice results in proteinuria and increases blood suPAR levels. **(B)** Replacement with BM cells from healthy mice significantly reduces suPAR levels and subsequently improves renal function in proteinuric mice. **(C)** The removal of uPAR-expressing IMCs prior to transfer of BM cells from proteinuric to healthy mice protects mice from proteinuria.

Unsurprisingly, these reports on the implication of suPAR as an FSGS factor soon generated excitement and follow-up studies (31, 32). Case reports emerged showing that lowering circulating suPAR levels through plasmapheresis or immunoadsorption could reduce proteinuria in recurrent FSGS (33), making it an effective therapy for some transplant FSGS patients (34, 35). Conversely, transmission of elevated suPAR from a mother with FSGS to her child was correlated to the child being born with proteinuria (36). Morphologically, podocyte effacement could be closely linked to suPAR levels at the time of post-transplantation FSGS occurrence (37). In line with these clinical observations, our findings obtained from animal experiments support the causative correlation between suPAR levels and renal function (38). Using a series of experimental approaches, including bone marrow transplantation and adoptive cell transfer, we discovered that bone marrow (BM)-derived immature myeloid cells (IMCs) are likely a main source of circulating suPAR, thereby contributing to proteinuric kidney diseases. These findings, in agreement with early observations (39), suggest the functional contribution of BM to kidney function and BM-derived IMCs as the possible origin of circulating suPAR responsible for renal injury. As evidence, we have shown that suPAR-generating cells transferred from proteinuric mice are essential for the induction of proteinuria and a concomitant suPAR increase in healthy mice (38) (Figure 1).

Particularly, in the first 5 years after the initial discovery of suPAR as a FSGS factor, not all studies with suPAR have resulted in supporting conclusions. This could be attributed to technical reasons such as differences in biological models or assays or a lack of power in some cohort-based studies. In a single center study of idiopathic FSGS in children, Bock et al. did not find the difference in serum suPAR levels among FSGS, non-FSGS glomerular disease, non-glomerular kidney disease and healthy controls (40). Subsequently, there were several studies from different groups questioning the usefulness of serum suPAR as a diagnostic marker for FSGS. The possibility of retention of suPAR due to decreased glomerular filtration rate (GFR) has been raised as well (41–44). The main argument was the relative unspecific nature of an elevated circulating suPAR, which could be observed in FSGS, but also in non-FSGS kidney disease and in many non-kidney disease. This discrepancy was clarified in a large cohort study establishing suPAR as a risk factor for chronic kidney disease (CKD) (45). Conflicting conclusions were arisen from different animal studies as well. We showed that the injection of full-length mouse recombinant suPAR protein (derived from NS0 cells, Fc Chimera, R&D systems) caused a mild proteinuria in uPAR knockout (*Plaur*^−/−^) mice. Whereas, Cathelin et al. did not detect proteinuria in wild type C57BL/6J mice injected with mouse full length suPAR protein purified from NS0 cells or a monomeric mouse full length uPAR isolated from eukaryotic S2 cells (46). In contrast to our experimental model where the secreted form of mouse uPAR was expressed via electroporation in wild type C57BL/6J mice (24), Spinale et al. utilized a full-length mouse suPAR transgenic floxed FVB mouse model. While the induction of mouse suPAR in the liver was achieved by retro-orbital injection of Adeno Associated Virus 8 particles that carry a hepatocyte-specific Cre recombinase, these mice did not develop proteinuria up to 44 days (47). These seemingly conflicting studies employed different models, methods and human patient cohorts, and If interpreted more carefully in their respective close context would have caused less confusion. As pointed out by late Schlondorff, these discrepancies should not discourage further research on the potential roles of suPAR in proteinuric kidney disease, including FSGS (48), and indeed they did not. In 2014, Delville et al. published a circulating antibody panel for pre-transplant prediction of FSGS recurrence after kidney transplantation. In their study, CD40 autoantibody alone had the best correlation (78% accuracy) with recurrent FSGS risk after transplantation; interestingly injection of CD40 autoantibody obtained from recurrent FSGS patients enhanced human suPAR-mediated proteinuria in wild type mice, suggesting the possible synergy between CD40 autoantibody and suPAR (49). Later, Alfano et al. found that full-length human suPAR down-regulated nephrin expression in human primary podocytes. Additionally they found infusion of this same human full length suPAR protein into uPAR knockout mice induces proteinuria (50). In individuals of recent African ancestry, variants in APOL1 have been associated with certain forms of CKD. In two large unrelated cohorts, Hayek et al. found that decline in kidney function associated with APOL1 risk variants is dependent on plasma suPAR levels. Their study suggested the synergy of circulating suPAR and APOL1 variant G1 or G2 on αVβ3 integrin activation is an underlying mechanism (51). Needless to say, the initial debate regarding suPAR and proteinuric kidney disease triggered and/or intensified the investigation of suPAR as a biomarker and risk factor for CKD and acute kidney injury (AKI), the details of which will be reviewed elsewhere.

To further understand the possible causative role of suPAR, we have created three different transgenic mouse models, constitutively expressing full-length mouse suPAR (muPAR1), secreted form of mouse suPAR (muPAR2) and mouse suPAR DIIDIII fragment under AP2 promoter, respectively. Compared to muPAR1, muPAR2 does not possess GPI anchor sequence and only have an intact DI. In certain experiments, the transgenic mice were fed with high fat diet to induce the expression of mouse suPAR. Interestingly, we observed different kidney pathologies with these transgenic mice: 4 months into high fat diet induction, muPAR1 transgenic mice developed significant low grade proteinuria in about one third of the mice (38); 6 months after high fat diet treatment, proteinuria became severe in some but not all muPAR1 transgenic mice (52). A small portion of muPAR1 transgenic mice developed spontaneous proteinuria by 1 year old without high fat diet treatment. In contrast, most muPAR2 transgenic mice developed spontaneous proteinuria by 2 months old without high fat diet. With high fat diet treatment, muPAR2 transgenic mice presented chronic and progressive proteinuria. By high fat diet induction for 6 months, some muPAR2 transgenic mice demonstrated a severe proteinuric kidney disease characteristic of FSGS changes. Mechanistically, msuPAR2 requires the presence of β3 integrin-Src signaling to generate proteinuria (52, 53). Collectively, these findings indicate that different forms of mouse suPAR generate kidney disease state with different severity, further reflecting the complexity of suPAR biology.

## suPAR/uPAR, More Than Just One Look

**Figure 2:**
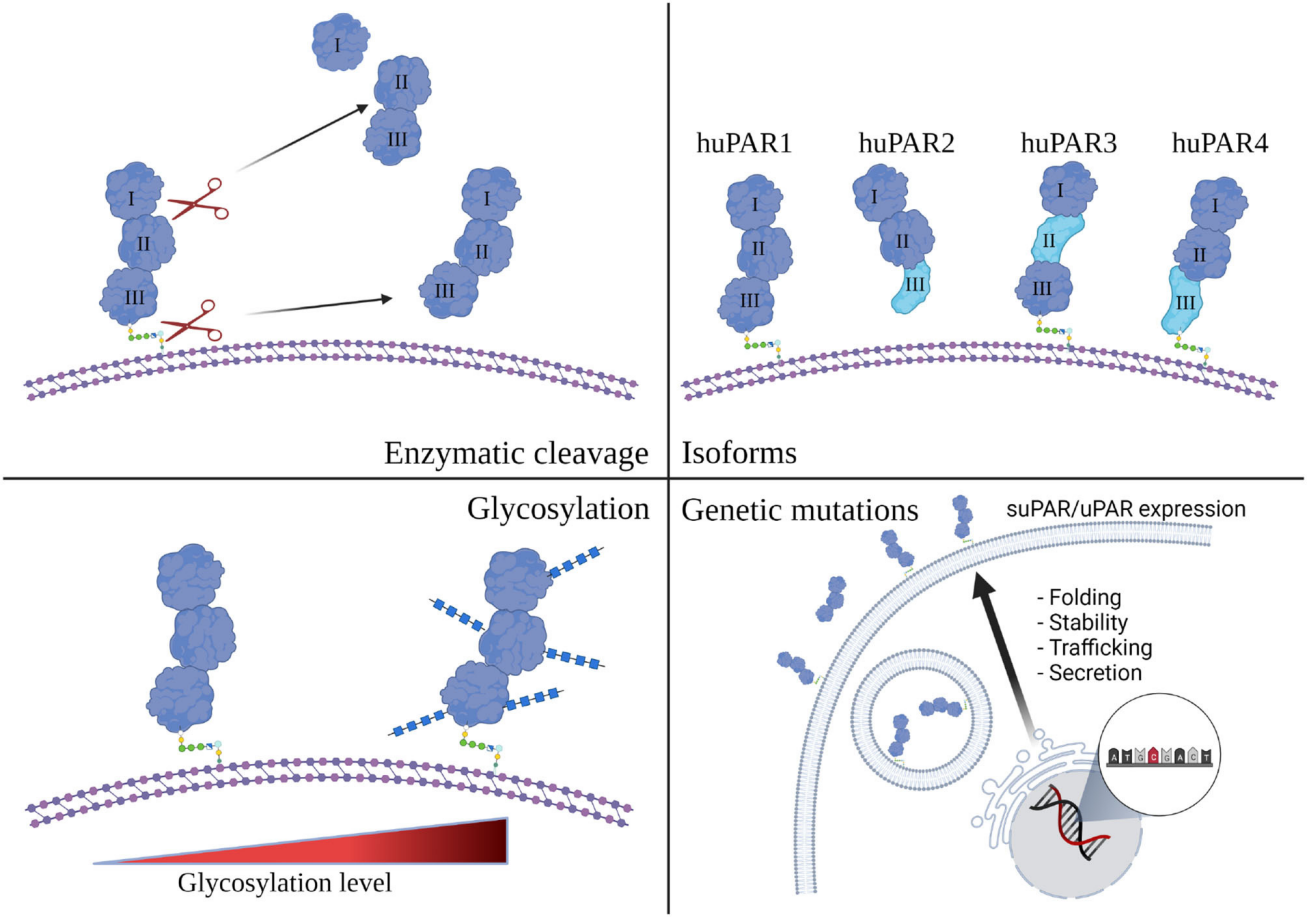
Human suPAR, more than just one flavor. Schematic presentation of human uPAR and suPAR (Created with BioRender.com). While enzymatic cleavage has been considered to be the main source of suPAR, transcriptional splicing, genetic mutation, and post-translational glycosylation could also impact the composition and function of circulating suPAR in different human subjects.

How can we understand the multifaceted role of suPAR in kidney disease? While generally known as suPAR, it clearly has more than one form. It has been documented that cleavage of GPI anchor releases full-length suPAR from membrane-bound uPAR (22). Numerous studies have indicated that full-length suPAR is functional (12). It retains uPAR's ability to bind to uPA, and suPAR binds vitronectin and integrins as well (9, 54, 55). As suPAR and uPAR can be cleaved at the linker region between DI and DII by a variety of enzymes (56), they generate DI fragment and DIIDIII fragment. Both fragments have been detected in body fluids (57, 58). DIIDIII fragment, while cannot efficiently bind uPA or vitronectin is active as it possess chemotactic properties shown by many studies (59–61). In addition to different suPAR fragments, there are other modifications that could impact circulating suPAR composition and function as well, including post-translational glycosylation, genetic mutation, and different isoforms derived from alternative splicing (Figure 2). The amino acid sequence for human uPAR/suPAR contains five N-linked glycosylation sites affecting the molecule mass and possibly the function of these proteins. Several different glycosylated variants have been reported in different cell types (62). Glycosylation profiling of a recombinant suPAR expressed in Chinese hamster ovary cells indicated that only four sites were utilized (63). How different glycosylated suPAR variants function in human body remains unknown. Recently, there are several studies presenting different uPAR genetic variants, correlating to circulating suPAR levels or not (64, 65). Understanding the role of these uPAR genetic mutations in human physiology and disease will be an exciting research avenue in the future.

## uPAR Isoform and Proteinuric Kidney Disease

Notably, uPAR has multiple isoforms both in human and in mice due to alternative splicing of the seven encoding exons (Table 1) (66, 67). About three decades ago, two alternatively spliced mouse uPAR mRNAs were identified in the gastrointestinal tract, with the full length, canonical form (muPAR1) localized in the luminal epithelial cells, and the shorter secreted form (muPAR2) found in the basal epithelial cells (66). Unlike muPAR1, which has 3 intact domains (DI to DIII) and 7 predicted sites of glycosylation, muPAR2 has only intact DI, encoded by exons 2 and 3, and part of DII as encoded by exon 4, missing the rest of the native protein (whole DIII and part of DII, encoded by exons 5–7), including GPI anchor. Judged from its amino acid sequence, msuPAR2 was once considered to be unstable due to its number of cysteine residues (68). We originally cloned muPAR2 mRNA (GenBank ID, BC010309) from cultured mouse podocytes, and found its protein interacting with integrin β3; once expressed in C57BL/6 mice via electroporation, muPAR2 induced proteinuric kidney disease (24). The pathogenesis of muPAR2 was later confirmed in muPAR2 transgenic mice that developed a chronic kidney disease, resembling FSGS. In addition, we purified msuPAR2 protein from HEK cells and characterized it as a stable protein, forming a dimer comprising DI and part of DII. The single long strand of β-sheet in the DII region might pair with the strand from its dimer partner (52). Our studies indicate that some form of suPAR causes FSGS-like changes at least in mice (24, 52).

**Table 1:** Major uPAR isoforms in mouse and human.

	**Isoform**	**Exon**	**Domains**	**GPI anchor**	**Length (Amino acid)**	**Nucleotide ID**	**Protein ID**
Mouse	Isoform 1 canonical form, muPAR1	7 Exons (1–7)	Three intact domains (I, II, III)	Yes	327	NM_011113	NP_035243
	Isoform 2 secreted, muPAR2	Exons 5 to 7 missing	DIII and part of DII missing	No	222	BC010309	CAA44575
Human	Isoform 1 canonical form, huPAR1	7 Exons (1–7)	Three intact domains (I, II, III)	Yes	335	NM_002659	NP_002650
	Isoform 2 secreted, huPAR2	Exon 7 missing	C-terminal part of DIII missing	No	281	NM_001005376	NP_001005376
	Isoform 3, huPAR3	Exon 5 missing	Part of DII missing	Likely	290	NM_001005377	NP_001005377
	Isoform 4, huPAR4	Exon 6 missing	N-terminal part of DIII missing	Likely	286	NM_001301037	NP_001287966

Compared to mouse uPAR, human uPAR has more splicing isoforms. So far, at least 4 major human uPAR isoforms have been documented in different human cells and tissues. While we detected these isoforms in human peripheral mononuclear cells (PBMC) by real-time quantitative PCR (52), Hagemann-Jensen et al. reported the presence of multiple uPAR isoforms in different T cells, monocytes and HEK cells by single cell RNA sequencing (69). Some of these uPAR isoforms could be detected in human glomeruli as well (personal communication with Dr. Mattias Kretzler from University of Michigan). Human isoform 1 (huPAR1) is equivalent to canonical muPAR1, with three intact Ly6/uPAR domains and a GPI anchor. Human isoform 2 (huPAR2) has a deletion of exon 7, and lacks a GPI anchor sequence. As with muPAR2, huPAR2 could provide the natural (secreted and uncleaved) source of suPAR. Human isoform 3 (huPAR3) has a deletion of exon 5, hence lacks the three C-terminal β-strands in DII. Human isoform 4 (huPAR4) has an in-frame deletion of exon 6, which contributes the N-terminal sheet assembly to DIII, but retains the 3 C-terminal strands of DIII and the GPI anchor. How do these splicing isoforms impact suPAR's composition and function remains unclear. Notably, the currently available ELISA kits are all utilizing different antibodies developed against huPAR1, the canonical form, yet they present different suPAR levels (70). While they can detect the full length human suPAR and DIIDIII fragments derived from huPAR1, they cannot efficiently detect most of alternative human uPAR isoforms.

What is the implication of different human uPAR isoforms in kidney? Since muPAR2 is associated with FSGS-like kidney changes in our mouse model, it is possible that overexpression of one or more of these human isoforms could be associated with the development of FSGS. Among these human uPAR isoforms, huPAR3 seems to be the closest to muPAR2 at least structurally. It likely forms the same dimer assembly as we observe in the msuPAR2 structure (52). Indeed, transient expression of huPAR3 in C57BL/6J mice induced proteinuria (unpublished data). In no doubt, further studies on alternative human uPAR isoforms are required to determine their respective roles in the pathogenesis in kidney disease.

In summary, elevation of suPAR is a circulating risk factor for kidney disease, including FSGS. Certain form of suPAR (i.e., muPAR2) causes FSGS-like changes in mice. The complexity of suPAR derived from different enzymatic cleavage, transcriptional splicing and post-translational modification may explain suPAR/uPAR's multifaceted roles. New technologies such as single cell based deep sequencing and proteomic analysis should help understand their respective underlying mechanisms in different disease settings. Detecting the different circulating uPAR isoforms in human samples could possibly provide differentiating diagnostic or prognostic value with different suPAR related disease.

## Author Contributions

CW, RS, EH, and JR wrote the manuscript. All authors contributed to the article and approved the submitted version.

## Funding

This work was supported by RO1DK125858, RO1DK109720, R01DK113761 (JR and CW), and RO1DK125394 (EH).

## Conflict of Interest

JR reports personal fees from Biomarin, Visterra, Astellas, Genentech, Merck, Gerson Lehrman Group, and Massachusetts General Hospital. He is the recipient of grants from Nephcure Kidney International and Thermo BCT. JR's lab is the recipient of fee-for-service funds from Walden Biosciences. JR is cofounder, scientific advisory board cochair, and shareholder of Walden Biosciences, a kidney therapeutic company. In addition, JR has the following patents: US20110212083, Role of soluble uPAR in the pathogenesis of proteinuric kidney disease; US9867923, Reducing soluble urokinase receptor in the circulation; JP2016530510, Non-glycoslyated suPAR biomarkers and uses thereof; US20160296592, Methods/compositions for the treatment of proteinuric diseases; US9144594, Dynamin mediated diseases; and US8809386, Dynamin ring stabilizers. EH is an inventor on a patent application of inducible costimulator ligand for use as a renal therapeutic. The remaining authors declare that the research was conducted in the absence of any commercial or financial relationships that could be construed as a potential conflict of interest.

## Publisher's Note

All claims expressed in this article are solely those of the authors and do not necessarily represent those of their affiliated organizations, or those of the publisher, the editors and the reviewers. Any product that may be evaluated in this article, or claim that may be made by its manufacturer, is not guaranteed or endorsed by the publisher.
